# The mediating effect of job involvement in the relationship between tennis Instructors' perceived organizational justice and turnover intentions: a multi-group analysis across generations

**DOI:** 10.3389/fspor.2024.1382751

**Published:** 2024-06-20

**Authors:** Sujin Kim, Jongho Kim

**Affiliations:** ^1^ Department of Sports Science Convergence, Dongguk University Seoul, Seoul, Republic of Korea; ^2^ Department of Sports Culture, Dongguk University Seoul, Seoul, Republic of Korea

**Keywords:** tennis instructors, turnover intentions, organizational justice, job involvement, MZ generation, multigroup structural equation modeling

## Abstract

**Introduction:**

This study aimed to analyze the role of job involvement as a mediator in the relationship between tennis coaches' perceived organizational justice and their intention to leave, considering the unique professional context and demands of tennis coaching. Additionally, it sought to identify any generational differences in this model. The research categorizes perceived organizational justice into procedural and distributive justice, and job involvement into job attachment and job commitment.

**Methods:**

The study incorporated data from 201 coaches working at commercial tennis facilities nationwide. Perceived organizational justice and job involvement were measured using validated scales. The mediation model was tested using structural equation modeling (SEM), and a multi-group analysis was conducted to identify generational differences.

**Results:**

Results indicated that job involvement partially mediated the relationship between perceived organizational justice and turnover intentions, with distributive justice having a stronger total effect. The multi-group analysis revealed generational variances: distributive justice influenced turnover intentions more among the MZ generation, while procedural justice had a greater impact on the older generation.

**Discussion:**

These findings offer valuable insights for commercial tennis facilities aiming to reduce turnover and manage generational conflicts. Understanding the differential impacts of procedural and distributive justice on various generations can help tailor strategies to enhance organizational operation and employee retention.

**Conclusion:**

The study highlights the importance of perceived organizational justice and job involvement in influencing tennis coaches' turnover intentions. The generational differences observed suggest that targeted interventions based on generational characteristics can be effective in reducing turnover and improving organizational stability. Future research should explore other potential mediators and extend the model to different sports and organizational contexts.

## 1 Introduction

In the wake of the COVID-19 pandemic that emerged in 2020, the global sports landscape underwent significant transformations, and South Korea was no exception to these changes. As a response to the pandemic, social distancing policies were implemented across the country, impacting various aspects of daily life, including access to sports facilities. However, a noteworthy phenomenon emerged amidst these challenges: the resilience and adaptability of the sports community. Remarkably, despite the constraints imposed by social distancing measures, the participation rate in physical activities in South Korea exhibited a remarkable resilience. The 2022 national survey revealed a notable 0.4% increase in regular sports participation compared to the previous year, signifying the enduring commitment of individuals to maintain an active lifestyle (1). Among the diverse array of sports, tennis emerged as a beacon of popularity, capturing the attention of enthusiasts and novices alike (2).

Tennis's surge in popularity can be attributed to several pivotal factors, particularly under the unique circumstances brought about by the pandemic. Notably, the spread of indoor tennis facilities emerged as a key solution to the pandemic-induced challenges. While outdoor activities were not banned, the use of public sports facilities was halted, and limitations were placed on the number of participants that could engage in exercise within the same space. This situation significantly influenced recreational preferences, steering individuals towards sports like tennis that could be practiced safely and individually. Indoor tennis, in particular, became highly appealing, providing athletes with the means to continue their sport in all weather conditions, within the safety and privacy of controlled environments. The distinct four-season climate of South Korea further underscored the advantage of indoor tennis, offering a stable and predictable setting for year-round participation. Additionally, the integration of advanced technology in these facilities elevated the training and playing experience to meet participants' demands for quality sports engagement (2). The availability of professional coaching and structured programs significantly contributed to skill development, intensifying the appeal of tennis. Following the pandemic, there was a noticeable rise in user loyalty, as these facilities adapted to meet the changing needs of tennis enthusiasts. This increase in satisfaction not only substantially boosted the sport's popularity but also led to a significant rise in the number of individuals learning and engaging in tennis. Consequently, the popularity of indoor tennis facilities catalyzed a trickle-down effect, naturally extending the increase in tennis participation to more traditional forms of the sport. This broader engagement has further cemented tennis as one of the preferred sports activities in South Korea, showcasing its widespread appeal across different playing environments.

However, this surge in popularity has not been without its challenges. One prominent issue that has surfaced is the high turnover rates among tennis instructors in the country (3). The rapid growth of indoor tennis facilities has resulted in an increased demand for skilled instructors, leading to a shortage in supply (3, 4). The tennis industry insiders have identified this issue as being intricately linked to evolving standards of perceived justice in employment treatment. As the status of instructors improved and their contributions became more recognized, so too did their expectations regarding fair and equitable treatment in the workplace. Compounding this issue is the prominence of the Millennial and Generation Z (MZ) generations among tennis instructors. Studies have consistently highlighted disparities in the perceptions of organizational justice between MZ and older generations, influencing the factors that drive turnover intentions among these instructors. These generational differences in values, expectations, and workplace preferences have added complexity to the challenge of retaining quality tennis instructors.

In light of these developments, this study sets out to comprehensively investigate the intricate relationship between organizational justice and commitment among tennis instructors. It aims to uncover how these factors collectively influence the intention to leave the profession, with a specific focus on comparing the dynamics between the MZ generation and their older counterparts. By delving into these dimensions, this research endeavors to offer practical insights that can be leveraged to address the issue of high turnover rates among tennis instructors in commercial tennis facilities. Moreover, this study aspires to contribute to a broader discourse within the field of sports organizational management. Beyond addressing the immediate concerns of the tennis industry, it seeks to offer holistic solutions that have relevance to wider issues facing sports organizations. By offering a nuanced understanding of the dynamics at play, this research has the potential to facilitate the effective management of generational conflicts within organizations, transcending its immediate scope.

In conclusion, the multifaceted dynamics of the COVID-19 pandemic have ushered in a new era in the world of sports, with tennis emerging as a symbol of resilience and adaptability. However, as the sport's popularity surges, challenges such as high instructor turnover rates have come to the forefront. This study endeavors to uncover the underlying factors at play and provide actionable insights to address these challenges while contributing to the broader discourse on sports organizational management.

## 2 Theoretical background

### 2.1 Turnover intention and influencing factors of tennis coaches

Turnover intention refers to the degree of will among employed members of an organization to sever their employment relationship and move to another organization (5, 6). Managers need to prevent turnover in advance by reducing the turnover intentions of key members for efficient organizational operation. Turnover can lead to severe organizational problems, such as gaps in roles and wasted administrative time to readjust work schedules (7). Especially in commercial sports organizations where demand is skyrocketing, reducing coaches' turnover intention is crucial for organizational operation.

In particular, turnover in commercial tennis facilities can have serious implications for organizational operation due to frequent replacement of coaches, necessitating an analysis of the factors influencing turnover. Related studies have analyzed the factors influencing turnover from dimensions such as organizational justice, job burnout, job satisfaction, organizational commitment, and leadership (7–9). Comprehensive prior studies analyzing the relationship between factors influencing the turnover intention of employees in sports-related facilities in Korea indicate that organizational justice (10, 11) organizational situation and job environment (12, 13), job commitment (14), and job satisfaction and burnout (15) influence turnover intention. Thus, this study sets perceived organizational justice and job commitment as key factors influencing turnover intention, based on these prior studies. Specifically, we propose a theoretical model in which job commitment mediates the relationship between organizational justice and turnover intention.

### 2.2 Organizational justice

Organizational justice refers to the degree to which members of an organization perceive themselves as being treated fairly and reasonably compared to other members (12). According to social comparison theory, employees deem they belong to a fair organization when they perceive that they receive appropriate rewards comparative to their abilities or position after assessing themselves against other employees (13). Organization justice conceptually comprises distributive and procedural justice (12, 14). Distributive justice relates to the concept of whether rewards such as within-group compensation, rights, and positions are reasonably allocated based on personal investment or ability. Procedural justice relates to the concept of how fair the criteria are in the process of deciding the distribution and whether the dispute resolution process is appropriate (16). The concept of organizational justice, composed of these two dimensions, is being analyzed as an important independent variable affecting the performance or efficient operation of an organization in human resource management, regardless of the field (14).

Prior studies related to justice in sports organizations commonly report that individuals who perceive injustice within the organization not only experience a decline in self-esteem but also a weakened commitment and trust in the organization, which increases their willingness to leave the organization (17, 18). On the contrary, it is reported that organizational members who perceive their organization as fair show higher job satisfaction and commitment (10). Moreover, several studies have distinguished the concepts of procedural justice and distributive justice to analyze the impact each may have on turnover intention (11, 19). Hence, it can be predicted that employees of sports organizations with a higher perceived organizational justice would experience higher job commitment, and that heightened job commitment would play a positive role in reducing turnover intention.

### 2.3 Job involvement

Job involvement refers to an organization member's belief that they can realize significant values in their life through their work, thus strongly immersing themselves in their duties (20). Typically, members of an organization who dedicate their best efforts to their assigned tasks and hold a benevolent attitude toward their duties tend to experience strong immersion. Schaufeli, Bakker, and Salanova (20) suggest that strong job involvement arises from two axes—attachment to one's work and commitment to the organization. According to their research, employees with high job attachment and a dedicated attitude to their work believe that their jobs hold a significant place in life's values, and they tend to strive for job excellence throughout their lives. Numerous prior studies suggest that organization members experiencing strong job involvement tend to experience job satisfaction, self-actualization, leading to a decrease in turnover intentions (21).

Previous studies analyzing the relationship between job involvement and turnover intentions among sports instructors suggest that the professional expertise developed during their career influences job involvement. This involvement, in turn, differentiates their turnover intentions. Moreover, through job involvement, these instructors build self-esteem and experience a high level of organizational commitment, further reducing their intention to leave (22, 23). Tennis instructors require a high level of expertise, and switching to other industries may be challenging. Thus, considerable time and effort are required to maintain their expertise. From this perspective, job involvement serves as a reward for the time and effort tennis instructors invest in acquiring their professionalism. It is therefore predicted that tennis instructors with a high level of job involvement would have lower turnover intentions due to the rewards provided by job involvement.

### 2.4 Generational differences in factors influencing turnover intentions among tennis coaches

People of the same age group typically share similar socio-economic experiences and cultural backgrounds, which implies that age and generation are significant predictors of individual attitudes, behavioral patterns, and socio-cultural perceptions (24). Each distinctive generation shares common values, which are primarily shaped by experiences from adolescence to early adulthood (24). The so-called “MZ Generation”—the millennials born between the early to mid-1980s and 2000, and Generation Z born in the early to mid-2000s—are acclimated to digital environments, favor “fun” and “convenience”, value individual happiness over group prosperity, sharing over ownership, balance between work and leisure (“work-life balance”), and are sensitive to fair returns on their efforts and investments (25, 26). Recent research has shown that the work values and attitudes shared by the MZ Generation differ from older generations, impacting the formation of unique organizational cultures (25, 26).

Organizational culture calls for a harmony between each member's job satisfaction and organizational commitment. Yet, achieving appropriate management in workplaces that span multiple generations can be challenging. Commercial sports facilities comprise instructors with various generational, background, and experience diversity, creating control difficulties (27). According to prior research, the majority of recreational tennis coaches have no specific ranking and often work under the same conditions irrespective of age and experience. This situation compels leaders from different generations, who pursue different job values, to perform identical tasks under the same remuneration system (28). In such an organizational culture, leaders from different generations pursuing varied values can perceive discrimination, potentially leading to conflicts between generations and operation teams (29).

Conversely, numerous studies have suggested that tennis coaches, who are satisfied and professional in their roles, exhibit strong leadership, resulting in participant satisfaction and sustained engagement (30). Therefore, fostering a healthy organizational culture in tennis facilities with diverse generations is crucial, from both organizational management and customer satisfaction perspectives. Satisfying user preferences is fundamental for the smooth operation of commercial tennis facilities, and this is shaped by the organizational environment, the quality of the coaches, and services, underlining the importance of creating a positive organizational culture (30).

Hence, to encourage tennis coaches, who offer the core services in commercial tennis facilities, to experience job satisfaction and commitment, it is necessary to explore the generational differences in factors influencing their turnover intentions. This exploration will equip operators with insights for efficient management and allow workers to expand their understanding of different generations, making this type of research essential. From this perspective, this study seeks to investigate how the relationships between perceived organizational justice, job commitment, and turnover intentions differ across generations.

RQ1: How does the relationship between turnover intentions among recreational tennis coaches and their perceived justice and job commitment present itself?
H1-1: The higher the perceived procedural justice, the greater the job attachment.
H1-2: The higher the perceived procedural justice, the greater the job dedication.
H1-3: The higher the perceived procedural justice, the lower the turnover intentions.
H1-4: The higher the perceived distributive justice, the greater the job attachment.
H1-5: The higher the perceived distributive justice, the greater the job dedication.
H1-6: The higher the perceived distributive justice, the lower the turnover intentions.
H1-7: The greater the job attachment, the lower the turnover intentions.
H1-8: The greater the job dedication, the lower the turnover intentions.
RQ 2: How do the relationships between turnover intentions, perceived justice, and job commitment among recreational tennis coaches differ between the MZ Generation and established generations (Generation X, Baby Boomers)?

## 3 Method

### 3.1 Research subjects

This study aimed to analyze the factors influencing the turnover intentions of recreational sports instructors working in the tennis education service industry and investigate the differences between the MZ generation and established generations. For this, a survey was conducted with 201 instructors working at indoor and outdoor tennis establishments from July 25, 2022, to August 4, 2022. Specifically, the clustering sampling method was used to select 201 tennis coaches working at indoor and outdoor tennis courts and sports facilities providing tennis lessons nationwide. To ensure the selection of a representative sample, this study engaged tennis instructors at facilities registered with the Korea Tennis Association (KTA) and the Korea Indoor Tennis Association (KITA), utilizing a combination of telephone and in-person surveys. This approach allowed us to not only affirm the study's objectives and obtain informed consent verbally but also facilitate participation through both online and paper surveys, accommodating the preferences of our respondents. The choice to focus our research on tennis facilities and instructors in South Korea was driven by the desire to address specific challenges emerging within this context—such as generational coexistence in the workforce and the rising demand for tennis instructors leading to job burnout. These issues, while presently pronounced in South Korea, possess broader implications, potentially affecting various societies and sports disciplines in the future. Our comprehensive methodology and focus on South Korea's tennis education sector aim to provide insights that are not only relevant locally but also applicable to similar challenges globally, enhancing the study's relevance and applicability. For the survey, calls and direct visits were made to 65 indoor tennis courts and 22 outdoor tennis courts. As a result, 112 field responses and 89 online responses were ultimately collected and used for analysis. Our study utilized digital surveys via Google Forms to ensure respondent convenience and anonymity, finding no significant differences in response quality compared to traditional paper surveys, thus validating the effectiveness of digital data collection methods.

Detailed information on the research subjects is presented in (Table 1).

**Table 1:** General characteristics of the study subjects.

	Category	Frequency	Percentage (%)
Gender	0: Male	155	77.11
	1: Female	46	22.89
Generation	1: Baby Boomers (1955–1963)	21	10.45
	2: Generation X (1970–1980)	81	40.30
	3: Millennial Generation (1981–1996)	79	39.30
	4: Generation Z (1997-)	20	9.95
Tenure	1: Less than 1 year	0	0.00
	2: 1 year to 3 years	19	9.45
	3: 3 years to under 5 years	28	13.93
	4: 5 years to under 7 years	62	30.85
	5: 7 years and over	92	45.77
Former athlete status	0: Athlete	159	79.10
	1: Non-athlete	42	20.90

### 3.2 Measurement variables

The respondents completed the survey using a self-administration method. Measurement tools presented in previous research were adapted to suit this study, aiming to measure the turnover intention and the factors influencing turnover of tennis instructors engaged in recreational sports. All independent variables, with the exception of employment type, were measured using Likert's 7-point scale (1: strongly disagree, 7: strongly agree).

To measure turnover intention, three items were constructed by referencing the “Turnover Intention Scale” developed by (5). This scale has been cited in over 651 academic studies since its recent development, which suggests it has sufficient external validity. For organizational justice, procedural justice and distributive justice were each measured using three items suggested by Colquitt (14) and Chung, Jong Sam (30). The respective scales are suitable for measuring the concept of justice in treatment among organization members, which was conceptually utilized in this study. Job involvement was measured by constructing three items related to job attachment and three items related to commitment, referencing Schaufeli et al. (20). Detailed items are presented in Table 2.

**Table 2:** Measurement items by variable.

Factor		Measurement	Reference
Dependent Variable	Turnover Intention	1. Even with similar working conditions, I hope to transfer to a different establishment within the same industry.	Bothma, Roodt (5)
		2. I am investigating employment opportunities at different establishments in the same industry.	
		3. I am willing to accept a job offer from a different establishment in the same industry.	
Organizational Justice	Procedures	1. Our establishment has procedures to determine the basis for rewards for each organizational member.	Colquitt et al. (31); Jung (32)
		2. Our establishment uses procedures that are fair in making compensation decisions.	
		3. The promotion procedures at our establishment are transparent and equitable.	
	Distribution	1. I have received fair compensation for the effort I put into my work.	
		2. I have received appropriate compensation that matches my contribution.	
		3. Compared to others, I have not received unreasonable compensation.	
Job Involvement	Attachment	1. I feel happy when I am working.	Schaufeli et al. (20)
		2. The work I do is a very important part of my self-actualization.	
		3. I intend to continue building my career in this industry.	
	Commitment	1. Even if the compensation were the same, I would not change to a different industry.	
		2. I would continue to work in this industry even if I had sufficient wealth.	
		3. I take pride in the work that I perform.	

### 3.3 Measurement variables

In order to validate the research hypotheses and research questions that were previously presented, AMOS 28 was utilized for conducting descriptive statistical analysis, measurement model validity verification, and multi-group structural equation analysis. Descriptive statistical analysis involved confirming the appropriateness of the data used in the analysis by examining the mean, standard deviation, skewness, kurtosis, and correlation among independent variables, along with item reliability analysis.

Subsequently, a confirmatory factor analysis was conducted to determine if the observed variables adequately explained the latent variables. In this study, the mean value of the sub-item measurement values was used to measure a single independent variable.

Convergent validity was confirmed using Composite Reliability (CR), and discriminant validity was ascertained using Average Variance Extracted (AVE). After verifying the appropriateness of the measurement variables for the analysis, a structural equation model encompassing parameters was validated.

In the following step, a multi-group structural equation model analysis was performed to scrutinize the differences between the MZ generation and the established generation. The significance of the difference in path coefficients derived from each model was verified, providing insights into the factors affecting the intention to leave.

## 4 Results

### 4.1 Descriptive statistics, correlation, and reliability analysis of variables

Table 3 presents the descriptive statistics for the primary variables measured in this study. All variables are continuous, and the mean, standard deviation, minimum, maximum, skewness, and kurtosis of each were examined. Observations on the mean values of the constructs revealed the following: turnover intention averaged at 3.94 (SD = 1.14), procedural justice at 4.10 (SD = 1.40), distributive justice at 4.50 (SD = 4.48), job attachment at 4.09 (SD = .91), and job commitment at 4.07 (SD = .89). To verify the normality of the main variables, skewness and kurtosis values were examined. Skewness ranged from .19 to.97 and kurtosis ranged from 1.03 to 2.52, indicating conformity to the normal distribution (33).

**Table 3:** Descriptive statistics of variables.

	Mean	Standard deviation	Minimum	Maximum	Skewness	Kurtosis
Turnover Intention	3.94	2.14	1	7	.25	2.28
Procedural Fairness	4.10	1.40	1	7	.97	2.52
Distributive Fairness	4.50	.99	1	7	.54	1.32
Job Attachment	4.09	.91	1	7	.19	1.03
Job Commitment	4.07	.89	1	7	.22	1.90

Table 4 presents the correlation analysis results for the main variables in this study. If the correlation coefficient between constructs to be used in multiple regression analysis exceeds .7, multicollinearity can occur, leading to statistical errors. However, the correlation coefficients among the main variables of this study were all less than .6, suggesting that there were no issues with statistical errors due to multicollinearity.

**Table 4:** Analysis of correlation between independent variables.

	Procedural fairness	Distributive fairness	Job attachment	Job commitment	Turnover intention
Procedural Fairness	.890	–	–	–	
Distributive Fairness	.632*	.901	–	–	
Job Attachment	.530*	.450**	.854	–	
Job Commitment	.531**	.433**.	.578*	.869	
Turnover Intention	.399***	.510***	.490***	.411***	.885

* *p* < 0.5.

** *p* < .01.

*** *p* < .001.

### 4.2 Verification of reliability and validity of measurement tools: CFA, reliability and validity test

To verify the reliability and validity of the measurement tools used in this study, a Cronbach's alpha test and confirmatory factor analysis were conducted. Notably, as the reliability and validity of the measurement tools can vary depending on the subject, this study confirmed whether the reliability and validity of the tools measured for each group (not only the whole group but also MZ generation and established generation) were satisfactory.

The results of the confirmatory factor analysis for the goodness-of-fit of the measurement model (Table 5) showed that the overall model [*χ*^2^ = 146.092 (df = 80, *p* < .01), *χ*^2^/df = 1.826, TLI = .957, CFI = .967, RMSEA = .051, SRMR = .059], the MZ generation model [*χ*^2^ = 136.307(df = 80, *p* < .01), *χ*^2^/df = 1.703, TLI = .946, CFI = .951, RMSEA = .059, SRMR = .062], and the established generation model [*χ*^2^ = 140.798(df = 80, *p* < .01), *χ*^2^/df = 1.759, TLI = .947, CFI = .954, RMSEA = .057, SRMR = .059] all appeared to be at a satisfactory level.

**Table 5:** Reliability analysis of measurement items (results of confirmatory factor analysis).

Factor	*b*	S.E	*t*	α			C.R			AVE		
				Total	MZ	Older generation	Total	MZ	Older generation	Total	MZ	Older generation
Turnover Intention 1	1.000	–	–	.886	.883	.891	.865	.864	.833	.792	.752	.703
Turnover Intention 2	.877	.025	27.29									
Turnover Intention 3	.643	.022	20.89									
Procedural Fairness 1	1.000	–	–	.889	.881	.908	.847	.738	.760	.743	.667	.691
Procedural Fairness 2	1.242	.035	22.36									
Procedural Fairness 3	.801	.020	19.85									
Distributive Fairness 1	1.000	–	–	.893	.903	.891	.833	.723	.761	.745	.692	.681
Distributive Fairness 2	1.022	.046	30.11									
Distributive Fairness 3	1.054	.051	19.91									
Job Attachment1	1.000	–	–	.917	.912	.905	.874	.752	.884	.727	.683	.725
Job Attachment2	.855	.035	20.49									
Job Attachment3	1.211	.022	18.89									
Job Commitment1	1.000	–	–	.901	.905	.891	.835	.834	.803	.742	.672	.688
Job Commitment2	.753	.031	22.25									
Job Commitment3	.668	.061	15.55									

Note 1. Total Model Fit: *χ*^2^ = 146.092(df = 80, *p* < .01), *χ*^2^/df = 1.826, TLI = .957, CFI = .967, RMSEA = .051, SRMR = .059.

Note 2. Model Fit for Generation MZ: *χ*^2^ = 136.307(df = 80, *p* < .01), *χ*^2^/df = 1.703, TLI = .946, CFI = .951, RMSEA = .059, SRMR = .062.

Note 3. Model Fit for the Older Generation: *χ*^2^ = 140.798(df = 80, *p *< .01), *χ*^2^/df = 1.759, TLI = .947, CFI = .954, RMSEA = .057, SRMR = .059.

As a result of conducting the reliability analysis, the alpha reliability values for each sub-area were between .886–.917 for the whole group, .880–.912 for the MZ generation, and .888–.918 for the established generation. As the reliability level can be considered satisfactory if it is above .6, the internal reliability of the items used in this study was found to be reliable.

To verify the convergent validity of the measurement tools, the Composite Reliability (CR) values were between .824–.891 for the whole generation, .723–.864 for the MZ generation, and .745–.884 for the established generation. Considering that the generally accepted figure is .5, the measurement tools of this study were found to be at an appropriate level (34). The Average Variance Extracted (AVE) values for verifying the discriminant validity of the measurement tools were between .694–.792 for the whole generation, .667–.752 for the MZ generation, and .676–.743 for the established generation. Referring to the commonly accepted figure of .5–.95, it was confirmed that the measurement tools of this study would secure discriminant validity (35).

### 4.3 The mediating role of organizational commitment in the relationship between organizational justice and turnover intention: structural equation modeling (RQ1)

In this study, a structural equation model analysis was executed to analyze the relationship between perceived organizational justice, job involvement, and turnover intention among participating sports tennis instructors. Based on the hypotheses (Hypothesis 1–1 to 1–6) of Research Question 1 previously presented, we constructed and analyzed the research model. The appropriateness of the research model was primarily analysed, and the results indicated *X*^2^(df) = 202.08(82), TLI = .929, CFI = .933, RMSEA = .053. This demonstrates that the structural equation model is at an appropriate level for interpretation (35).

**Figure 1:**
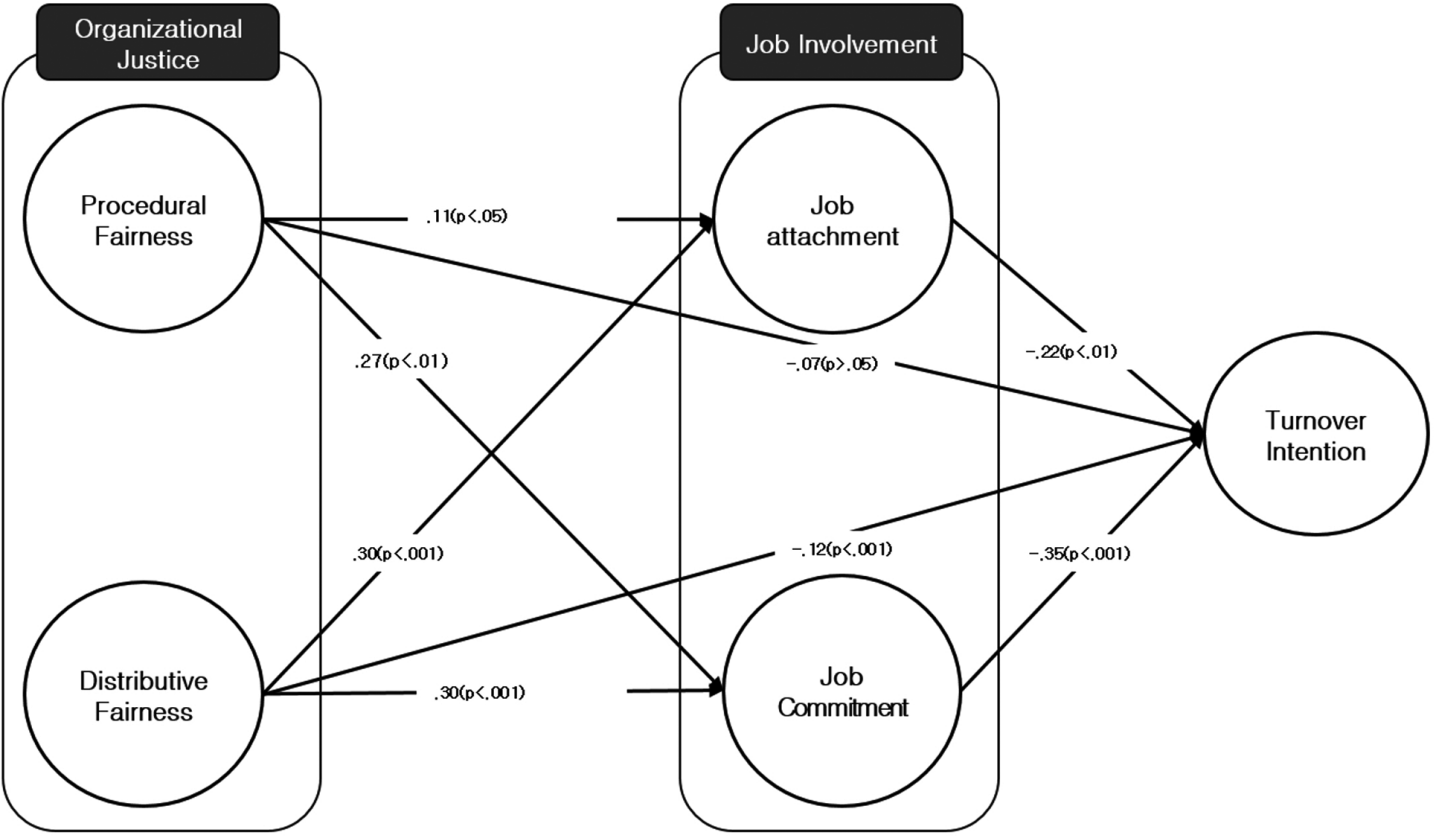
Structural equation model (RQ1).

Upon a detailed examination of the path process of the structural model in (Figure 1), it was observed that procedural justice and distributive justice of organizational justice affect turnover intention through two paths—job attachment and job commitment of job involvement. Procedural justice has a positive effect on job attachment (*b* = .10, *p *< . 05) and job commitment (*b* = .27, *p* < .01), and both job attachment and job commitment have a negative impact on turnover intention. Also, perceived distributive justice significantly has a positive effect on job attachment (.30, *p* < .01) and job commitment (.30, *p* < .001). Consequently, it was confirmed that organizational justice has a positive influence on job involvement and job involvement impacts reducing turnover intention. Moreover, the impact of distributive justice on job attachment and job commitment was found to be greater than that of procedural justice, and job commitment has a greater impact on reducing turnover intention than job attachment.

In addition, the mediating effect of job involvement mediating the impact of organizational justice on turnover intention was verified through a decomposition of effects analysis (Table 6). Specifically, the indirect effects of job attachment and job commitment mediating the impact of procedural justice on turnover intention and those mediating the impact of distributive justice on turnover intention were analyzed respectively. The direct impact of procedural justice on turnover intention was .07, and the indirect impact was .119. The significance of the mediating effect for these results was verified by checking the significance of the indirect confidence interval using bootstrapping, which was found significant as it does not include 0 in the 95% confidence interval. Thus, it was found that job attachment and job commitment partially mediate the relationship between procedural justice and turnover intention. The direct impact of distributive justice on turnover intention was .12, and the indirect impact was .075. The significance of the mediating effect for these results was verified by checking the significance of the indirect confidence interval using bootstrapping, which was found significant as it does not include 0 in the 95% confidence interval. Thus, it was found that job attachment and job commitment partially mediate the relationship between distributive justice and turnover intention. Therefore, the partial mediating effect of job involvement in the relationship between organizational justice and turnover intention was validated.

**Table 6:** Mediation analysis (effect decomposition).

Path	Direct effect	Indirect effect	Total effect	Indirect confidence interval (bias corrected, 95%)
Procedural Fairness → Turnover Intention	−.07	.119	.189	.011–.057***
Distributive Fairness → Turnover Intention	−.12	.075	.175	.019–.062***

* *p* < .05.

** *p* < .01.

*** *p* < .001.

### 4.4 Verification of generational differences in the turnover intention model of tennis instructors: multi-group structural equation analysis (RQ2)

We conducted a multi-group structural equation analysis after dividing the generations into MZ and established generations to identify the generational differences in the factors influencing the turnover intentions of instructors working at lifestyle sports facilities. In order to compare the path coefficients of the two groups, the analysis was conducted by comparing the unconstrained model and the constrained model through the form invariance and structure invariance between the two models. As shown in (Table 7), the unconstrained model, which is a form invariance model, was at a good level with *χ*^2^ = 455.822 (df = 174), CFI = .903, and RMSEA = .049. The measurement invariance model, which constrained the path between latent variables and observed variables, showed a relatively good fit between the model and the data with *χ*^2^ = 590.112 (df = 194), CFI = .912, RMSEA = .047, but it did not show a statistically significant difference from the form invariance model in the difference of *x*^2^. Through this, it was confirmed that the observed variables measuring each constituent concept were perceived identically between groups. The structure invariance model, which constrained the variance and covariance of latent variables, was analyzed as *χ*^2^ = 601.331 (df = 210), CFI = .911, RMSEA = .043, and through this, it was confirmed that the model and the data were showing a relatively good fit. In addition, it was confirmed through the statistically significant difference in the comparison of the *x*^2^ difference between the form invariance and the measurement invariance model that the groups distinguished by generation had an effect as a moderator.

**Table 7:** Comparison of model Fit between unconstrained and constrained models.

Model	*χ* ^2^	df	CFI	RMSEA	Δ*χ*^2^	Sig
Configural Invariance	455.822	174	.903	.049	–	–
Metric Invariance	590.112	194	.912	.047	10	.058 (rejected)
Structural Invariance	601.331	210	.911	.043	4	.022 (accepted)

**Figure 2:**
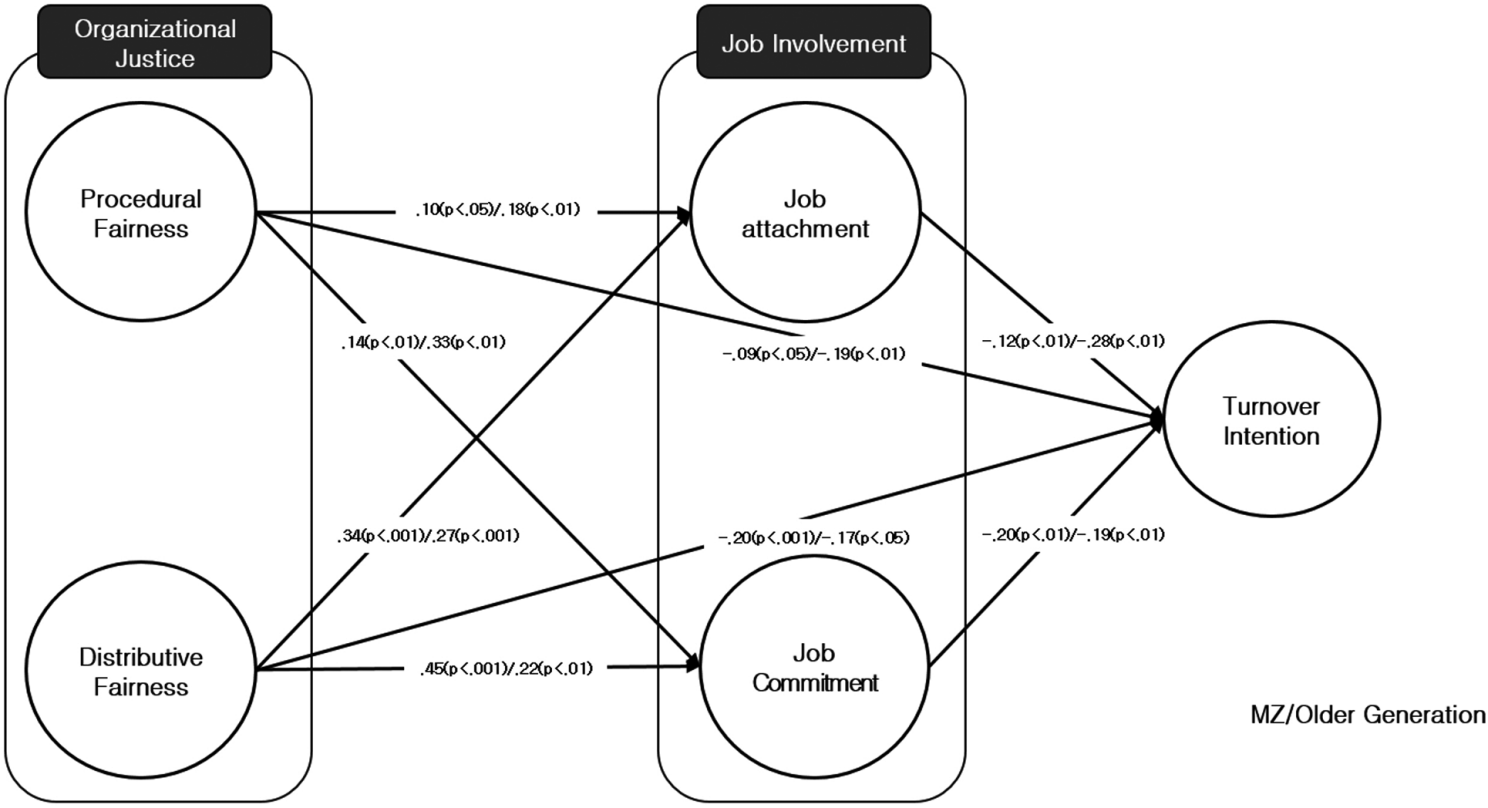
Multi-group structural equation model (RQ2).

Based on these results, we conducted a path coefficient comparison analysis between the generational groups. The analysis results are as follows in (Table 8 and Figure 2). First, looking at the path process of the MZ generation structural model in detail, in the case of procedural justice, it had a significant impact on job attachment (*p* = .10, *p* < .05), job commitment (*p *= .14, *p* < .01), and turnover intention (*p* = −.09, *p* < .05). Distributive justice also had a significant impact on job attachment (*p* = .34, *p* < .001), job commitment (*p* = .45, *p* < .001), and turnover intention (*p* = −.20, *p* < .01). Job attachment (*p* = −.12, *p* < .01) and job commitment (*p* = −.20, *p* < .01) had a significant impact on turnover intentions.

**Table 8:** Estimation and comparison of path coefficients across groups.

Path	Path coefficient difference (Δ*χ*^2)^	MZ		Older generation	
		*b*	S.E	*b*	S.E
Procedural Fairness → Job Attachment	1.12	.10*	.100	.18**	.094
Procedural Fairness → Job Commitment	**3**.83**	.14**	.090	.33**	.093
Procedural Fairness → Turnover Intention	0.93	−.09*	.104	−.19**	.090
Distributive Fairness → Job Attachment	**3**.09*	.34***	.099	.27***	.106
Distributive Fairness → Job Commitment	**4**.03**	.45***	.092	.22**	.124
Distributive Fairness → Turnover Intention	0.81	−.20***	.081	−.17*	.101
Job Attachment → Turnover Intention	2.02	−.12**	.093	−.28***	.089
Job Commitment → Turnover Intention	0.99	−.20*	.092	−.19**	.096

Bold numbers indicate statistically significant levels.

* *p* < .05.

** *p* < .01.

*** *p* < .001.

As a result, in the MZ generation model, organizational justice had a positive impact on job involvement, and job involvement influenced reducing turnover intentions. Also, distributive justice had a greater impact on job attachment and job commitment than procedural justice, and job commitment had a greater impact on reducing turnover intention than job attachment. Then, in the effect decomposition analysis conducted to verify the mediating effect of job involvement that mediates the impact of organizational justice on turnover intentions, the direct effect of procedural justice on turnover intentions was −.094, the indirect effect was −.041, and the total effect was −.135. The direct effect of distributive justice on turnover intentions was −.206, the indirect effect was −.103, and the total effect was −.309. The significance of the mediating effects of these two results was confirmed by checking the significance of the indirect confidence interval using bootstrapping, and all were confirmed to be significant as they did not include 0 in the 95% confidence interval. As a result, it was confirmed in the MZ generation model that job involvement partially mediates.

## 5 Conclusion and discussion

The aim of this study was to analyze the influence of organizational justice factors, such as perceived procedural justice and distributive justice, on turnover intention among participatory sports tennis instructors, along with the mediating effects of job attachment and commitment. Moreover, the study aimed to examine the generational differences between MZ generation and established generations. To achieve these objectives, we established the research model and hypothesized relationships based on previous studies. We applied multi-group structural equation modeling to validate the mediating effects. The principal findings and their implications are discussed as follows.

### 5.1 Intention, organizational justice, and job involvement among tennis instructors

In Study 1 of this research, we conducted a structural equation analysis based on a research model developed using preceding research and theories, aimed at analyzing the mediating role of job involvement in the relationship between tennis instructors' turnover intention and organizational justice. The results supported all Hypotheses 1–1 to 1–8, and partial mediating effects of job involvement were validated. In this study, we distinguished organizational justice into procedural justice and distributive justice, and job involvement into job attachment and job commitment, then interpreted the meanings by analyzing the differences in path coefficients. The following are the summaries and discussions of the main results of Study 1.

First, both procedural justice and distributive justice played positive roles in job attachment and commitment. However, it was confirmed that distributive justice had a greater impact on both job attachment and commitment than procedural justice. This tendency was also found in the relationship with turnover intention. From this, we could infer that task volume and salary determination could play important roles in turnover intention. More than 60% of the tennis instructors participating in this survey were contracted under a salary system that set salaries based on the amount of teaching or employment contracts that paid incentives based on task volume after meeting the basic task volume. Therefore, we can infer that the lower the turnover intention, the more appropriately the salary paid per hour or per class is determined. Shon et al. (28) suggested that for sports instructors to feel stable in their jobs, the pay system must be stable. Therefore, a reasonable determination method for task volume and pay system is needed to reduce the turnover intention of participatory sports tennis instructors and to make them feel stable in their jobs.

Second, the indirect effect of procedural justice on turnover intention through job attachment and commitment was greater than its direct effect, indicating that job involvement plays an important mediating role. In this case, job commitment had a greater impact on the relationship between procedural justice and turnover intention than job attachment. Previous studies have suggested that providing fair opportunities and democratizing the decision-making process play a positive role in increasing procedural justice, and establishing a performance evaluation and reward system based on clear criteria plays a positive role in increasing distributive justice (17, 18). Therefore, setting clear work goals and expectations for instructors, recognizing, and encouraging their performance, and constructing an educational system within the organization where continuous personal development can take place, all play a positive role in enhancing job commitment and attachment, respectively.

Third, the path coefficients of the factors directly affecting turnover intention appeared in the order of job commitment, job attachment, distributive justice, and procedural justice. It is notable that in the model established in this study, the factor that had the most significant impact on turnover intention was job commitment, which was higher than the total effect of procedural justice and distributive justice on turnover intention. This suggests the importance of policies and programs that promote job commitment within the organization. Job commitment refers to instructors having a strong identity and attachment to the organization, as well as support and commitment to the goals and values of the organization. Ultimately, the higher the satisfaction with their job, the longer they stay in the organization, and the lower their turnover intention. This aligns with the results of many previous studies on the role of job involvement in preventing organizational deviance (22). That is, tennis instructors can enhance job commitment by not only strengthening their capabilities through performing their duties in the organization but also fostering strong relationships and a sense of belonging to the organization.

### 5.2 Turnover intention, organizational justice, and job involvement among tennis instructors in the MZ generation and established generation

In Study 2 of this research, we performed a multi-group analysis to explore the differences in the relationship model between turnover intention, organizational justice, and job involvement for tennis instructors in the MZ generation and the established generation, a model previously analyzed in Study 1. The following is a summary and discussion of the key findings of Study 2.

Firstly, significant differences were observed between the MZ generation and the established generation in the relationship between perceived organizational justice, turnover intention, and job involvement of participatory sports tennis instructors. The model invariance analysis using comparisons between constrained and unconstrained models for multi-group structural equation modeling showed satisfactory levels of form, measurement, and structural invariance, indicating that the structure model of job involvement mediating the relationship between organizational justice and turnover intention differs in the MZ and established generations. Such findings allow us to infer the cognitive characteristics of the MZ generation that distinguishes work and organization.

Secondly, while the MZ generation placed significant importance on job attachment, they comparatively placed less importance on commitment to the organization. On the other hand, the established generation valued organizational commitment as much as job attachment. Schaufeli et al. (20) argued that job involvement is formed when these two aspects are fulfilled, subsequently reducing turnover intentions. The results of this study illustrate that the patterns suggested in previous research can differ across generations. In the field of tennis instruction, instructors are not usually in permanent employment contracts, and given that their work is physically demanding service provision, their career lifespan is not typically lengthy. Most instructors do not form deep connections with the organization. Therefore, for the MZ generation of tennis instructors with shorter careers, it can be interpreted that while a sense of responsibility or attachment to organizational tasks may form, attitudes of personal devotion may not.

Thirdly, organizational justice had a significant impact on turnover intention in both the established and MZ generations, with no intergenerational differences discovered. Both procedural justice and distributive justice were found to significantly influence turnover intentions in both generations, implying that perceived organizational justice plays a crucial role in shaping turnover intention, regardless of the generation of tennis instructors. According to Lambert & Hogan (7), when clear task divisions are not established at work, job burden is easily felt, leading to a sensitive response to fair task distribution. Tennis instructors have heavy workloads, providing sports education and various information about the sport to students while simultaneously handling membership registrations and schedule management. Thus, the finding of this study that turnover intention is formed based on the degree to which instructors perceive that the organization's task distribution and reward system are fair, regardless of generation, can be interpreted as aligning with previous research.

Lastly, in the case of the MZ generation, distributive justice had a greater impact on turnover intention than procedural justice, while in the established generation, procedural justice had a greater influence than distributive justice. This can be attributed to the notion that the perception and understanding of justice differ somewhat between generations. Ahn and Lee (8) proposes that the MZ generation, compared to the established generation, values individual happiness over group happiness, prefers sharing over ownership, and naturally expects rewards based on effort rather than experience. In contrast, the established generation has a high degree of organizational identification and equates their success with that of the organization, attributing more meaning to their lives than the MZ generation. Interpreting these findings in the context of the results of this study, we can conclude that operational policies in tennis facilities should differ according to the generation of the instructor. That is, for the MZ generation who values distributive justice, incentives should be provided for tasks outside of coaching. For the established generation, who values procedural justice, fair procedures for task division, promotion, and role allocation must be guaranteed to reduce turnover intentions.

### 5.3 Discussion

The results of this study are meaningful in that they provide crucial information for the organizational management dimension of commercial sports facilities. Recently, in recreational sports facilities of popular sports like tennis, problems such as the lack of instructors, disruption of work involvement and job burnout among organization members, and conflicts between the established generation and the incoming MZ generation have been highlighted. For commercial sports facilities to operate smoothly in line with the boom in recreational sports, it is essential to prevent the departure of organizational members who provide core services and to manage various factors that influence turnover intention rationally. To do this, it is necessary to identify factors that have a general level of influence, as well as to devise a subtle operating strategy through the identification of influence factors that differ by generation. The distributive justice and procedural justice suggested as results of this study can be considered as influence factors that administrators can respond to immediately. Moreover, the result of this study that justice and job attachment, which influence the turnover intention of the MZ and established generations, are differentially expressed, provides practical information that there needs to be differentiation in the way administrators understand and manage organizational members.

“2–1”ond the practical implications, the outcomes of this study hold academic significance in that they extend the theoretical scope related to factors influencing turnover, traditionally followed in organizational management, by employing the concept of organizational justice. Furthermore, by utilizing job attachment and commitment to leverage a multi-mediator effect, our research systematically refines the theoretical model. Notably, by focusing on the differences in perceptions of fairness across generations within the sports industry workplace, and exploring the factors influencing turnover intentions, our study showcases its academic uniqueness. As previously discussed, instructors who deliver learning to participants using physical exertion face not only mental but also significant physical consumption in job burnout. umerous studies have approached preventing job burnout from aspects such as working conditions, environment, compensation, employment type, contractual conditions, and the assurance of holidays. However, despite its critical importance, research on the specificity of the sports environment where different generations perform the same tasks, and on the conflicts between the zeitgeist represented by the MZ generation and the established generations, is a rarity within sports organization management studies. Therefore, this study holds academic value as a cohort study that explores sports management within the broader conceptual framework of organizational management, illuminating the unique context of the sports environment and investigating generational differences.

Despite these points, this study has several limitations. We propose the following suggestions for future research based on specific limitations. Firstly, there is a limitation to generalization because the data was only collected from workers at tennis facilities. As the specificity of facilities can also differ depending on the sport, if clusters are classified more sophisticatedly based on sport and facility, and a multilevel model type of research design is conducted accordingly, more valid, and empirical results are expected. Secondly, there was insufficient consideration of general control variables such as gender and salary. Differences can be shown even in the same work environment and job according to gender, and attitudes towards work can change depending on salary. However, there was a limitation that the gender ratio of the research subjects was imbalanced to introduce gender as a variable, and many were non-regular workers, so it was impossible to determine an average salary. In future research, if these points are supplemented and a research model designed well with control variables is suggested, it is expected that more reliable research results can be derived. Lastly, this study had limitations in that it verified variables derived from prior research, not exploratory variables reflecting the complex situation of the current recreational sports industry, which influence turnover intention. A deep exploratory process is required to reflect the current social situation and derive more practical variables. In future research, if qualitative research or research that includes exploratory variables is conducted based on the results of this study, it is expected that more empirical evidence can be established for factors that influence turnover and generation-specific tendencies.

## Data Availability

The raw data supporting the conclusions of this article will be made available by the authors, without undue reservation.
